# The Antiviral Effect of Nirmatrelvir/Ritonavir during COVID-19 Pandemic Real-World Data

**DOI:** 10.3390/v15040976

**Published:** 2023-04-16

**Authors:** Vasilios Petrakis, Petros Rafailidis, Grigorios Trypsianis, Dimitrios Papazoglou, Periklis Panagopoulos

**Affiliations:** 1 Department of Infectious Diseases, 2nd University Department of Internal Medicine, University General Hospital Alexandroupolis, Democritus University Thrace, 68132 Alexandroupolis, Greeceppanago@med.duth.gr (P.P.); 2 Department of Medical Statistics, Medical School, Democritus University of Thrace, 69100 Komotini, Greece

**Keywords:** SARS-CoV-2, COVID-19, nirmatrelvir/ritonavir, antiviral treatment, Omicron variants

## Abstract

Introduction: Vaccination against SARS-CoV-2 and the prevalence of Omicron variants have reduced the risk of the severe clinical progress of COVID-19. However, the risk of breakthrough infections has increased, and early administration of an effective antiviral treatment is significant in order to prevent the severe progression of COVID-19 in vulnerable patients with comorbidities. Patients and methods: Adults with confirmed SARS-CoV-2 infection were included in a matched-pair retrospective study based on age, gender, comorbidities and vaccination status. They were divided into two groups: group A (n = 200) consisted of outpatients at increased risk of severe clinical progress who were treated with nirmatrelvir/ritonavir and group B (n = 200) consisted of non-hospitalized patients who did not receive antiviral treatment. Demographic data, clinical outcome (death, intubation), days of hospitalization, time for recovery, adverse events and treatment compliance were reported. Results: The median age (75.24 ± 13.12 years in the study group and 76.91 ± 14.02 years in the comparison group) and the proportion of males (59% vs. 60.5%, respectively) were similar between the two groups. A total of 6.5% of patients in group A and 10.5% in group B were unvaccinated against SARS-CoV-2. Three patients from group A (1.5%) and one hundred eleven (55.5%) from group B required hospitalization. The duration of hospitalization (3 days vs. 10 days in group B, *p* < 0.001) and the total time needed for recovery (5 days vs. 9 days, *p* < 0.001) was shorter in the study group. A rebound of SARS-CoV-2 infection within 8–12 days after diagnosis was documented in 6.5% of patients in group A and 8% of patients in group B. Conclusion: Oral treatment with nirmatrelvir/ritonavir in high-risk non-hospitalized patients was safe and effective in preventing the severe clinical progress of COVID-19 pneumonia. Early administration of antiviral agents in vulnerable outpatients combined with a full vaccination scheme is significant in order to avoid hospitalization and severe clinical outcomes.

## 1. Introduction

COVID-19, which is caused by severe acute respiratory syndrome coronavirus 2 (SARS-CoV-2), remains a serious global health problem [1]. Primary and boosted vaccination against SARS-CoV-2 has reduced the risk of severe clinical progress [2]. In the era of Omicron variants, the rates of intubation and death have been reduced [3]. However, the risk of breakthrough infections has increased [4]. A number of treatment options have been evaluated in order to prevent the severe progression of COVID-19 in vulnerable patients with comorbidities such as immunosuppression, cardiovascular disease, diabetes mellitus, chronic renal disease, malignancies, pulmonary disease, and obesity [5].

Remdesivir (GS-5734), an inhibitor of the viral RNA-dependent RNA polymerase, is the first approved antiviral agent for hospitalized patients with COVID-19 pneumonia proven to lead to a shorter period of recovery and prevent progression to severe respiratory disease [6]. Although studies have shown that a 3-day course of remdesivir in non-hospitalized patients at high risk resulted in a lower risk of hospitalization or death, its usage in outpatients is limited due to the need for intravenous administration [7,8]. Treatment with monoclonal antibodies has been discontinued as it also requires intravenous or subcutaneous administration in a health care setting and is not effective against Omicron variants due to mutations in the monoclonal antibody-targeted SARS-CoV-2 spike protein [9]. Thus, oral antiviral agents that retain their efficacy, such as molnupiravir and nirmatrelvir/ritonavir, are widely administered.

Molnupiravir is the isopropyl ester prodrug of the ribonucleoside analogue β-D-N4-hydroxycytidine (NHC) [10]. In vitro studies showed that molnupiravir is a potent inhibitor of SARS-CoV-2 replication with an EC50 in the submicromolar range and leads to decreased time for viral RNA clearance [11]. Molnupiravir had shown promising efficacy and safety in phase I/II/III clinical trials, reducing the risk of hospitalization or death by approximately 50% in non-hospitalized adults with mild-to-moderate COVID-19 disease who were at risk of a severe clinical outcome [12]. The incidence of drug-related adverse events was low [12]. However, further studies showed than molnupiravir achieved only a 30% reduction in COVID-19-related hospitalization or death 29 days after randomization, and thus its use has been restricted to patients with end-stage renal disease who cannot receive alternative antiviral agents [13].

Nirmatrelvir (PF-07321332) is an oral antiviral agent that targets the SARS-CoV-2 3-chymotrypsin-like cysteine protease enzyme (Mpro) [14]. Mpro is essential in the viral replication cycle mainly because it enables nonstructural protein subunits to be cleaved off the polyprotein and rearranged [14]. As an antiviral target, it has a low likelihood of off-target activity due to the absence of recognized human analogues [14]. Nirmatrelvir is metabolized mainly by CYP3A4 and is co-administered with a low dose (100 mg) of ritonavir, a CYP3A4 inhibitor, as a booster [15]. Co-administration with ritonavir retards the metabolism of nirmatrelvir in order to remain active at high concentrations for a longer period [15]. Studies evaluating the safety and efficacy of nirmatrelvir plus ritonavir in non-hospitalized adults with mild-to-moderate COVID-19 at high risk have shown an 89% lower risk of severe clinical outcome [16]. Nirmatrelvir/ritonavir received emergency-use approval for the treatment of non-hospitalized COVID-19 patients at high risk of severe disease who did not require supplemental oxygen in January 2022 [17]. A few adverse events have been reported, mainly from the gastrointestinal system (dysgeusia, diarrhea, and vomiting) [18]. A clinical review of potential contraindications with concomitant drugs is crucial when the co-administration of ritonavir with medications dependent on CYP3A metabolism may result in significantly elevated concentrations of those drugs or in reduced nirmatrelvir concentrations that limit treatment efficacy [18,19].

The aim of the present study is to evaluate the safety and efficacy of nirmatrelvir/ritonavir in preventing severe clinical progress in high-risk outpatients based on the Guidelines of COVID-19 Treatment of National Public Health Organization published on 17 October 2022 [20]. This study includes real-world data, and the efficacy of nirmatrelvir/ritonavir is evaluated in a matched-pair study regarding age, gender, comorbidities and vaccination status. Real-world data are vital in order to evaluate the impact of nirmatrelvir/ritonavir and determine strategy policies for the treatment of vulnerable populations based on the epidemiological data for circulating variants, the risk of rebound phenomenon and the vaccination coverage. Our study analyzes the effect of nirmatrelvir/ritonavir treatment on 28-day recovery, hospitalization, intubation and mortality among outpatients during a SARS-CoV-2 Omicron (BA.2, BA2.12.1, BA.4, and BA.5)-predominant period in Greece.

## 2. Patients and Methods Objectives, Patients

This was a retrospective study conducted in the Clinic of Infectious Diseases of the University General Hospital of Alexandroupolis (Greece). Data from routine care patient charts during the period from 1 March 2022 to 1 March 2023 were retrospectively analyzed. The study was carried out in accordance with the Helsinki Declaration of Human Rights. The study evaluated the effectiveness and safety profile of treatment with nirmatrelvir/ritonavir among non-hospitalized adults with confirmed SARS-CoV-2 infection at high risk of progression to severe disease. Patients included in the study were at least 18 years old, had confirmed SARS-CoV-2 infection, experienced symptom onset no more than 5 days before drug administration and had at least one coexisting condition associated with a high risk of progression to severe COVID-19. Patients with anticipated hospitalization within 48 h, severe drug interactions with concomitant medications and/or those being treated with alternative antiviral agents were excluded.

## 3. Procedures

Adults with confirmed SARS-CoV-2 infection were included in a matched-pair study. Patients were classified into two groups. Group A (study group) consisted of increased risk, non-hospitalized patients who received nirmatrelvir/ritonavir orally. The dosing scheme was 300 mg nirmatrelvir and 100 mg ritonavir twice daily for 5 days, or 150 mg nirmatrelvir and 100 mg ritonavir twice daily when the estimated glomerular filtration rate was 30–59 mL/min per 1.73 m^2^. Tablets were to be administered with or without food and be swallowed whole. Antivirals were dispensed to the public through hospital pharmacies according to the national guidelines for SARS-CoV-2 antiviral treatment. Patients with a positive result from a real time-polymerase chain reaction (RT-PCR) or rapid antigen test (RAT) and risk factors of progression to severe disease were eligible for administration of nirmatrelvir/ritonavir after the approval of physicians, which was requested using an electronic platform during the first 3 days of symptom onset or after a positive test. Risk factors for progression to severe disease were defined by the guidelines of National Public Health Organisation and included immunosuppression due to disease or medication (including HIV-positive status with <200 CD4), malignancies, chronic renal disease, chronic respiratory disease, cystic fibrosis, age ≥ 75, age > 65 plus a chronic comorbidity, age < 65 plus 2 chronic comorbidities [20]. Group B (comparison group) consisted of non-hospitalized patients with SARS-CoV-2 infection during the same period of time/who did not receive oral antiviral agents due to unwillingness or drug interactions without available treatment modification for comorbidities or delayed clinical estimation after 5 days from symptoms onset. Patients of group B were treated based on the National Guidelines for hospitalized patients with COVID-19 pneumonia if hospitalization was needed [20].

## 4. Efficacy

The number of days from symptom onset until the first dose of treatment, the presence of respiratory failure and the clinical outcome (intubation, death) were reported. Medication adherence for nirmatrelvir/ritonavir based on the number of pills missed was documented. Demographic data, vaccination status and previous SARS-CoV-2 infection were also analyzed. Possible drug interactions of nirmatrelvir/ritonavir with concomitant drugs and their management were reported. Concomitant medications with potential interactions with nirmatrelvir/ritonavir were evaluated in order to determine appropriate therapeutic options, including temporary disruption, dose adjustment, or continuation with increased monitoring. The primary endpoint of the study was hospital admission or death within 30 days after a positive SARS-CoV-2 test. As secondary endpoints were evaluated during intensive care unit (ICU) admission, the need of mechanical ventilation and/or death within 60 days after the diagnosis of SARS-CoV-2 infection indicates more severe clinical progression.

## 5. Safety

Adverse events that emerged during or after the treatment period until day 28, serious adverse events, and adverse events leading to discontinuation were reported through the electronic platform and in the medical records of patients.

## 6. Statistical Analysis

Statistical analysis of the data was performed using the IBM Statistical Package for the Social Sciences (SPSS), version 19.0 (IBM Corp., Armonk, NY, USA). The normality of quantitative variables was tested using the Kolmogorov–Smirnov test. Normally, distributed quantitative variables are expressed as the mean ± standard deviation (SD), whereas non-normally distributed quantitative variables are expressed as the median value and range. Qualitative variables were expressed as absolute and relative (%) frequencies. Student’s *t*-test, the Mann–Whitney U test and the chi-square test were used to determine differences in demographic and clinical characteristics between the two groups of patients. All tests were two-tailed and *p* values < 0.05 were considered statistically significant.

## 7. Results

The present study was matched-paired with respect to age, gender and comorbidities. Each group consisted of 200 patients. The median age (75.24 ± 13.12 years in the study group and 76.91 ± 14.02 years in the comparison group) and the proportion of males (59% vs. 60.5%, respectively) were similar between the two groups. A total of 6.5% of patients in group A and 10.5% in group B were unvaccinated against SARS-CoV-2. The full vaccination scheme with two booster doses was completed in 10% of patients in group A and in 7% in group B, and 45% and 38% were vaccinated with one booster dose, respectively. Seven patients from group A (3.5%) and eight from group B (4%) received two vaccine doses within 6 months. More than 6 months after vaccination, 32.5% of group A and 38% of group B were reported to have received two doses. The percentage of individuals with previous SARS-CoV-2 infection was 8.5% in group A and 9% in group B. One patient in group A and three in group B were hospitalized again due to COVID-19 pneumonia. These four patients, during their hospitalization, had mild respiratory failure, radiologic findings of pneumonia and persistent fever for more than four days, but no patients were intubated. They were not hospitalized due to reinfection during our study. The most frequent comorbidities for group A and B were hypertension (64.5% vs. 68.5%), obesity (29.5% vs. 30.5%), diabetes mellitus (37% vs. 39.5%), heart failure (33% vs. 33.5%), atrial fibrillation (14.5% vs. 16%), malignancies (22% vs. 21%), chronic respiratory disease (8% vs. 9%) and immunosuppression (35.5% vs. 34.5%). More than two comorbidities (multi-comorbidities) were present in 68% of patients in group A and in 72% in group B with four coexisting comorbidities in 45% and 56%, respectively. Results are shown in Table 1.

Three patients from group A required hospitalization (1.5%). None of the patients were intubated or died. In group B, one hundred eleven (55.5%) patients were hospitalized, seventy-three (36.5%) had respiratory failure, six (3.0%) were intubated and nine (4.5%) died. The number of days from symptom onset until treatment initiation was significantly lower in group A (2 days vs. 7 days in group B, *p* < 0.001). The duration of hospitalization (3 days vs. 10 days in group B, *p* < 0.001) and the total time needed for recovery (5 days vs. 9 days, *p* < 0.001) was shorter in the study group.

Adverse events had occurred in 23 (11.5%) patients in the study group and were mild and self-limited (Table 2). The most common were stomachache (2.5%), nausea (4.5%), vomiting (2.5%) and dysgeusia (3.5%). No serious adverse events were documented and no disruption of treatment was needed. No lab abnormalities associated with nirmatrelvir/ritonavir were documented. Only two patients received treatment for adverse events, but no hospitalization or additional clinical visit was needed.

Partial treatment compliance based on the number of missing pills was reported in 2.5% of patients treated with nirmatrelvir/ritonavir (Table 2). Five patients discontinued their treatment two or three days after initiation due to significant clinical improvement despite medical advice to complete the 5-day treatment course. Rebound of SARS-CoV-2 infection with positive rapid antigen test and/or new symptoms onset within 8–12 days after diagnosis was documented in 6.5% of group A and 8% of group B. None of these patients was hospitalized while mild, self-limited symptoms for a short period of time occurred.

Among patients treated with nirmatrelvir/ritonavir, drug interactions with concomitant drugs for comorbidities were identified in 78 individuals (39%) which were clinically significant and required mitigation. The most common identified drug interaction was between 3-hydroxy 3-methylglutaryl-CoA reductase inhibitors and ritonavir (n = 58, 29%). Statins were not administered for 7 days after the initiation of antiviral treatment. Anticoagulants such as rivaroxaban, dabigatran and apixaban were replaced with low molecular weight heparin (LMWH) for 7 days (n = 16, 8%) and no events of bleeding or thrombosis were reported. Cardiovascular drugs such as calcium channel blockers (amlodipine, 4%), angiotensin II receptor blockers (valsartan, 6%) and non-dihydropyridines (diltiazem 2.5%, verapamil 2.5%) were continued with monitoring of blood pressure. Dose adjustment was performed if needed. A similar management strategy was followed for psychiatric drugs (haloperidol 1%, alprazolam 4%, risperidone 0.5%, diazepam 1%). Drugs for benign prostatic hyperplasia such as tamsulosin (3%) were discontinued for seven days. Drug interactions and their management strategies are shown in Table 3.

Multivariable logistic regression models for all patients (group A and B, n = 400) showed that treatment with nirmatrelvir/ritonavir (OR 0.34, 95% CI 0.29–0.55, *p* < 0.001) and a complete vaccination scheme against SARS-CoV-2 (OR 0.24, 95% CI 0.19–0.29, *p* < 0.001) were significantly associated with a lower probability of severe clinical progress of COVID-19 (Table 4). On the other hand, increased age (OR 2.78, 95% CI 2.65–2.85, *p* < 0.001), male gender (OR 2.04, 95% CI 1.95–2.29, *p* < 0.001) and comorbidities increase the risk for severe clinical outcome (Table 4). Malignancies (OR 3.65, 95% CI 1.54–3, *p* < 0.001) and immunosuppression (OR 5.73, 95% CI 4.84–7.14, *p* < 0.001) were the major comorbidities leading to hospitalization, intubation and/or death.

Multivariable logistic regression analysis regarding the effectiveness of treatment with nirmatrelvir/ritonavir based on treatment adherence showed that complete COVID-19 vaccination (two booster doses) (HR 0.14, 95% CI 0.09–0.18, *p* < 0.001), previous SARS-CoV-2 infection (OR 0.54, 95% CI 0.47–0.71, *p* < 0.001) and complete treatment adherence (0R 0.27, 95% CI 0.15–0.47, *p* < 0.001) increased the efficacy of the antiviral treatment (Table 5).

## 8. Discussion

The present study provides real-world data for the safety profile and efficacy of the orally administered antiviral agent nirmatrelvir/ritonavir in patients at high risk of severe clinical progression of COVID-19. The results of the study have indicated that adults treated with nirmatrelvir/ritonavir had more than 90% lower risk of hospitalization. Only three patients were hospitalized but an oxygen supplement was not required and no death or intubation occurred until day 28. The duration of hospitalization and time for recovery was significantly shorter for patients who received nirmatrelvir/ritonavir. Additionally, the possibility of a rebound of COVID-19 was found to be lower in group A. Patients of group B did not receive treatment with nirmatrelvir/ritonavir due to delayed medical advice and clinical evaluation or contradictions and drug interactions with concomitant drugs. The delayed hospitalization (median time 7 days after symptoms onset) in group B led to the limitation of therapeutic options.

Similarly with our study, the EPIC-HR trial (Evaluation of Protease Inhibition for COVID-19 in High-Risk Patients) in non-hospitalized adults with mild-to-moderate COVID-19 at high risk of progression to severe disease showed an 89.1% relative risk reduction in COVID-19-related hospitalization or death from any cause by day 28, regardless of age, sex, race, body mass index (BMI), baseline serology status, viral load, coexisting conditions, or number of coexisting conditions at baseline [16]. Subgroup analyses of the primary endpoint in this study showed that patients treated with nirmatrelvir plus ritonavir either had no COVID-19-related hospitalization or death from any cause or had a significantly lower risk than patients treated with a placebo [16]. In our study, only three patients treated with nirmatrelvir/ritonavir were hospitalized but none of them had respiratory failure, were intubated or died.

The EPIC-HR trial also indicated that nirmatrelvir/ritonavir leads to an additional reduction in SARS-CoV-2 viral load at day 5 [16]. An observational cohort study carried out by the First Hospital of Jilin University that included 258 patients treated with nirmatrelvir/ritonavir and 224 nontreated patients with mild-to-moderate COVID-19 at high risk of progression to severe disease showed that nirmatrelvir/ritonavir was associated with more rapid conversion from positive to negative SARS-CoV-2 RT-PCR compared with nontreated patients [21]. The median time for RT-PCR negative conversion was 7 days earlier in patients receiving treatment within 5 days after symptom onset [21]. However, patients who received treatment >5 days after symptom onset did not accelerate RT-PCR negative conversion, highlighting the significance of early administration of antiviral treatment [21]. Notably, a real-world study in Israel including 180,351 eligible patients, 4737 (2.6%) of whom were treated with nirmatrelvir/ritonavir and 135,482 (75.1%) of whom had adequate COVID-19 vaccination status, showed that treatment with nirmatrelvir/ritonavir in the first 5 days of SARS-CoV-2 infection was associated with a markedly reduced risk of progression to severe COVID-19 or mortality, regardless of vaccination status for SARS-CoV-2 [22]. Higher efficacy of nirmatrelvir/ritonavir was documented in older patients, immunosuppressed patients, and patients with underlying neurological or cardiovascular diseases [22]. Both the antiviral treatment and an adequate COVID-19 vaccination status were associated with a significant decrease in the rate of severe COVID-19 or mortality with adjusted HRs of 0.54 and 0.20, respectively [22]. Logistic regression analysis in our study concluded similar findings, with ORs of 0.34 and 0.24, respectively.

A number of studies based on real-word data have proven the effectiveness of nirmatrelvir/ritonavir [23,24,25]. In an observational study with data from the Hong Kong Hospital Authority during a wave of the Omicron subvariant BA.2.2, nirmatrelvir plus ritonavir use was associated with lower risks of death (0.34 [0.22–0.52]), hospitalization (0.76 [0.67–0.86]), and in-hospital disease progression (0.57 [0.38–0.87]) [24]. Another retrospective study in Hong Kong among outpatient and hospitalized patients with mild-to-moderate COVID-19 at high risk, early initiation of molnupiravir and nirmatrelvir/ritonavir treatment within 5 days of symptom onset was associated with a lower risk of in-hospital death and led to potential savings in health care costs [25]. An observational, retrospective cohort study based on data obtained from the electronic medical records of members of the Clalit Health Services (CHS), which covers approximately 52% of the entire Israeli population and almost two thirds of older adults, showed that during the Omicron surge, the rates of hospitalization and death due to COVID-19 were significantly lower among adults aged 65 years or older who had received treatment with nirmatrelvir than among younger adults, regardless of previous SARS-CoV-2 immunity [26]. Similar findings were reported in a published subgroup analysis of the EPIC-HR trial [16]. These data suggest that nirmatrelvir/ritonavir has no benefit for younger adults [16,26]. A metanalysis including a total of 13 studies and 186,306 patients showed that the overall OR for death and hospitalization among COVID-19 patients treated with nirmatrelvir/ritonavir compared with the control group was 0.22 (95% CI, 0.11–0.45; I 2 = 93%, *p* < 0.0001) [27]. Treatment with nirmatrelvir/ritonavir reduced the mortality or hospitalization rate by 78% [28]. Subtype analysis indicated an 88% reduction in mortality and a 68% reduction in hospitalization rate among COVID-19 patients treated with nirmatrelvir/ritonavir [29]. A comparative retrospective cohort study conducted in the USA using the TritNetx research network showed that administration of nirmatrelvir/ritonavir in vaccinated outpatients within five days of COVID-19 diagnosis led to a 45% relative risk reduction in the occurrence of subsequent emergency room visits, hospitalizations, or deaths compared to untreated patients [28]. Additionally, a reduced symptom burden and limited number of complications such as lower respiratory tract infection or cardiac arrhythmia were reported, whereas this benefit was not negated in treated patients who experienced a rebound of COVID-19 [28].

A prospective observational study conducted at the University Hospital of Pisa (January 2022–July 2022) included COVID-19 outpatients with at least one risk factor for disease progression who received nirmatrelvir/ritonavir, molnupiravir, or 3-day remdesivir treatment [29]. The risk of severe clinical progression did not differ among the three antiviral agents and only 2.5% of treated patients died or were hospitalized due to COVID-19 [29]. The frequency of adverse events with nirmatrelvir/ritonavir was high (49.2%) in this study, with dysgeusia being the most common [30]. In our study, the percentage of patients that reported adverse events was significantly lower (11.5%). The MOVe-OUT trial has shown that early use of molnupiravir within 5 days of symptoms onset reduced the relative risk of hospitalization or death by 30% and thus it is used only in patients with end-stage chronic renal failure [9]. Remdesivir has shown an 87% reduction in the risk of progression to severe disease in the outpatient setting but its use is limited due to the intravenous route of administration [7].

Studies assessing the in vitro antiviral activity of remdesivir, molnupiravir and nirmatrelvir have shown that they remain active against SARS-CoV-2 Omicron and other variants of concern and their target proteins (the viral RNA dependent RNA polymerase and the viral main protease Mpro) are highly conserved [31]. COVID-19 therapeutic monoclonal antibodies lost their efficacy against emerging variants due to mutations in the monoclonal antibody-targeted SARS-CoV-2 spike protein [32]. The Omicron variants have mutations in both the RNA-dependent RNA polymerase (RdRp) and the main protease of SARS-CoV-2, which are targets for antiviral drugs such as RdRp inhibitors (remdesivir and molnupiravir) and the main protease inhibitor PF-07304814, 5. Studies evaluating the efficacy of the three different antiviral compounds (i.e., remdesivir, molnupiravir, and PF-07304814) against Omicron showed that the in vitro 50% inhibitory concentration (IC50) values of each compound and the susceptibilities of Omicron to the three compounds were similar to those of the early strain [33].

SARS-CoV-2 variants managed to develop resistance to antibody-mediated neutralization and various therapeutic options lost their efficacy [34,35]. Thus, there is a concern that SARS-CoV-2 could also become resistant to nirmatrelvir [34,35]. Results of studies in both Vero E6 cells and Huh7-ACE2 cells demonstrate that in vitro high-level resistance to nirmatrelvir can be developed by SARS-CoV-2 with multiple pathways [34]. A combination of amino acid substitutions in 3CLpro (L50F E166A L167F) has been identified which is associated with a significant increase in 50% effective concentration (EC50) values for nirmatrelvir (PF-07321332) [35]. Substitutions E166A and L167F lead to low-level resistance, but the triple mutant with L50F achieved the highest levels of resistance in studies [35]. All substitutions can cause loss of enzymatic 3CLpro activity and reduce viral fitness [35]. According to the Paxlovid label, the mutation E166V is more common in nirmatrelvir/ritonavir-treated subjects compared to the placebo group (0.8% and <0.2%, respectively), and in one patient with pre-existing L50F substitution, the E166V substitution was developed on day 5 of treatment [34]. Studies including more than 480 performed independent selection experiments concluded that E166V confers the highest level of resistance of all substitutions tested (100× for nirmatrelvir) [36]. The absence of nirmatrelvir resistance in patients to date is due to the high drug concentrations achieved with the prescribed regimen and administration for a short period during activation of the immune system against the virus [35].

Some concerns were raised from case series that associated a rebound of SARS-CoV-2 infection with antiviral treatment [37]. These case reports document that some patients with a normal immune response who have completed a 5-day course of nirmatrelvir/ritonavir and then recovered can experience recurrent illness 2 to 8 days later with subsequent positive viral antigen and/or reverse transcriptase polymerase chain reaction (RT-PCR) testing [32,37]. The recurrence of illness and positive test results improved or resolved without additional anti-COVID-19 treatment [37]. In May 2022, the US Center for Disease Control and Prevention (CDC) issued a Health Alert Network Health Advisory in order to underline the potential for COVID-19 rebound after nirmatrelvir/ritonavir treatment [38]. However, studies including more than 10,000 patients showed no significant difference between the patients treated with nirmatrelvir/ritonavir and the control group in terms of COVID-19 rebound (OR = 0.84; 95% CI: 0.67–1.04, *p* = 0.11) [32,37]. In our study, the percentage of rebound COVID-19 was lower in outpatients receiving nirmatrelvir/ritonavir compared to untreated patients (6.5% vs. 8%, *p* = 0.004). These results indicate that the rebound phenomenon is not related to antiviral treatment but is a consequence of the viral infection. This rebound phenomenon could be the result of a secondary immune-mediated response after the completion of therapy leading to the resumption of SARS-CoV-2 viral replication and recurrence of clinical symptoms [39,40]. Studies have not concluded whether this rebound develops due to inadequate length of antiviral treatment or a natural biphasic pattern of the viral life cycle [41]. However, there are no data supporting significant effectiveness by administering longer courses or a second course of nirmatrelvir/ritonavir [39].

The limitations of this study are its retrospective nature and the limited number of patients included. Further studies across a larger patient series are needed in order to evaluate the findings of our study. Another potential limitation is that our study analyzed data retrospectively during the period from 1st March 2022 to 1 March 2023 without taking into consideration the different Omicron subvariants. BA.1* was the dominant subgroup until early March 2022, and BA.2* became the most prevalent until the end of May [42]. BA.2* was gradually replaced by BA.5* subgroup [42]. Additionally, there are only limited data for the different types of previous immunity of patients for both groups. The study included patients with previous immunity without reporting the time from previous infection and/or last dose of vaccination. Real-world studies have shown that treatment with nirmatrelvir/ritonavir is associated with a significantly reduced risk of progression to severe COVID-19 or mortality, regardless of vaccination status [22,43]. However, adequate vaccination status against SARS-CoV-2 remains the most effective intervention to prevent severe illness [22,43,44]. Our study showed that COVID-19 vaccine is independently associated with a significant decrease in the risk of severe COVID-19 disease (Table 4 and Table 5). The extensive vaccination status of patients in group A and B probably affected and enhanced the antiviral effects of treatment with nirmatrelvir/ritonavir.

In conclusion, oral treatment with nirmatrelvir/ritonavir in high-risk non-hospitalized patients was effective in preventing the severe clinical progress of COVID-19 pneumonia. Need of hospitalization was limited and no death or intubation was reported. Although vaccination, hybrid immunity and the prevalence of Omicron variants reduced the cases of severe pneumonia, early administration of antiviral agents in vulnerable outpatients combined with a full vaccination scheme is significant in order to avoid hospitalization and unfavorable clinical outcomes. This study has suggested that nirmatrelvir/ritonavir is a safe treatment option, but further studies are needed to assess the short- and long-term safety of this treatment method based on real-word data.

## Figures and Tables

**Table 1:** Demographic data, vaccination status, comorbidities and clinical characteristics of Group A (un-hospitalized patients treated with nirmatrelvir/ritonavir) and Group B (untreated patients with oral antivirals).

	Study Group Group A (n = 200)	Comparison Group Group B (n = 200)	*p* Value
Male gender, n (%)	118 (59.0)	121 (60.5)	0.654
Age Vaccination status	75.24 ± 13.12	76.91 ± 14.02	0.970
Single dose 2 doses (<6 months) 2 doses (>6 months) 1st booster dose 2nd booster dose Unvaccinated Previous SARS-CoV-2 infection	5 (2.5) 7 (3.5) 65 (32.5) 90 (45.0) 20 (10.0) 13 (6.5) 17 (8.5)	5 (2.5) 8 (4.0) 76 (38.0) 76 (38.0) 14 (7.0) 21 (10.5) 19 (9.5)	0.547 0.447 0.867 0.997 1.000 0.657 0.657
Comorbitidies			
ΒΜΙ >30	59 (29.5)	61 (30.5)	0.758
Hypertension	129 (64.5)	137 (68.5)	0.987
Diabetes mellitus	74 (37)	79 (39.5)	0.765
Heart Failure	66 (33)	67 (33.5)	0.831
Atrial Fibrilation	29 (14.5)	32 (16.0)	0.867
Ischemic heart disease	31 (15.5)	33 (16.5)	0.826
Neoplastic disease or hematologic malignancy	44 (22)	42 (21.0)	0.631
Respiratory problems	18 (9)	16 (8.0)	0.727
Immunosuppresion	71 (35.5)	69 (34.5)	0.413
Days of symptoms onset	2 (1–4)	7 (4–13)	<0.001
Hospitalization	3 (1.5)	111 (55.5)	<0.001
Days of hospitalization	3 (2–5)	10 (5–42)	<0.001
Respiratory Failure	0 (0.0)	73 (36.5)	<0.001
Intubation	0 (0.0)	6 (3.0)	0.034
Death	0 (0.0)	9 (4.5)	0.052
Time for recovery (days)	5 (3–11)	9 (5–18)	**<0.001**
Rebound Infection Yes	13 (6.5)	16 (8.0)	**0.004**

**Table 2:** Treatment adherence and adverse events in patients treated with nirmatrelvir/ritonavir (Group A, n = 200).

	Treated Patients (n = 200) n, (%)	*p* Value
**Treatment Adherence** Partial (missing pills) Total (no missing pills)	5 (2.5) 195 (97.5)	0.421 - -
** Adverse Events **		
Any adverse reaction	23 (11.5)	0.257
Adverse reaction above Grade 3	0	-
Treatment for adverse reaction	2 (1)	-
Discontinuation of treatment	0	-
Need for extended hospitalization	0	-
Additional clinical visit	0	-
Stomachache	5 (2.5)	-
Nausea	9 (4.5)	-
Vomiting	5 (2.5)	-
Dysgeusia	7 (3.5)	-

**Table 3:** Identified drug interactions with concomitant drugs for comorbidities and management strategy in patients treated with Nirmatrelvir/Ritonavir (Group A, n = 200, treated patients).

	Treated Patients (n = 200) n, (%)	Management
Any identified drug interaction	78/200 (39)	
**Cardiovascular drugs**		
Amlodipine Valsartan Diltiazem Verapamil	8 (4) 12 (6) 5 (2.5) 5 (2.5)	Continue, Monitoring Blood Pressure & Dose adjustment if needed
**Anticoagulants** (Rivaroxaban, Apixaban, Dabigatran)	16 (8%)	Stop for 7 days Administration LMWH *
**Statins** (Atorvastatin, Rosuvastatin, Simvastatin)	58(29%)	Stop for 7 days
** Psychiatric ** Haloperidol Risperidone Alprazolam Diazepam	2 (1) 1(0.5) 8 (4) 2 (1)	Continue. Monitoring Continue. Monitoring Dose adjustment. Monitoring Dose adjustment. Monitoring
** Benign Prostatic Hyperplasia ** Tamsulosin	6 (3)	Stop for 7 days

* LMWH, Low Molecular Weight Heparin.

**Table 4:** Logistic Regression Analysis for Severe COVID-19 (Hospitalization, Intubation, Death) (n = 400, Group A and B).

Variable	OR (95% CI)	*p* Value
**Nirmatrelvir/Ritonavir**		
Treated patients	0.34 (0.29–0.55)	<0.001
Untreated patients	2.54 (2.23-2.75)	<0.001
**COVID-19 vaccination**		
Complete scheme (2 booster doses)	0.24 (0.19–0.29)	<0.001
Incomplete scheme	1.03 (0.97–1.23)	<0.001
Unvaccianted	2.45 (2.36–2.88)	<0.001
**Age** (HR for each 10-year increase)	2.78 (2.65–2.85)	<0.001
**Male** sex	2.04 (1.95–2.29)	<0.001
**Comorbidities**		
Diabetes	2.07 (1.85–2.23)	<0.001
Cardiovascular disease	1.98 (1.63–2.19)	<0.001
Chronic lung disease	2.33 (1.75–2.81)	<0.001
Chronic kidney disease	2.56 (1.86–2.83)	<0.001
Malignancies (hematologic, solid)	3.65 (1.54–3.0)	<0.001
Immunosuppression	5.73 (4.84–7.14)	<0.001

**Table 5:** Logistic Regression Analysis for the efficacy of treatment with nirmatrelvir/ritonavir (prevention of hospitalization/intubation/death) based on treatment adherence (n = 200 Group A, partial compliance n = 5).

Variable	OR (95% CI)	*p* Value
** COVID-19 vaccination **		
Complete scheme (2 booster doses)	0.14 (0.09–0.18)	<0.001
Incomplete scheme	1.03 (0.97–1.23)	<0.001
Unvaccinated	1.25 (1.16–1.62)	<0.001
**Age** (HR for each 10-year increase)	1.78 (1.63–1.94)	<0.001
**Male** sex	1.04 (1.04–1.15)	<0.001
** Previous SARS-CoV-2 infection **		
Yes	0.54 (0.47–0.71)	<0.001
No	1.02 (0.97–1.12)	<0.001
** Treatment adherence **		
Complete	0.27 (0.15–0.47)	<0.001
Partial	1.12 (1.08- 1.56)	<0.001

## Data Availability

Not applicable.
